# Peer-Led Team Learning Helps Minority Students Succeed

**DOI:** 10.1371/journal.pbio.1002398

**Published:** 2016-03-09

**Authors:** Julia J. Snyder, Jeremy D. Sloane, Ryan D. P. Dunk, Jason R. Wiles

**Affiliations:** 1 Department of Biology, Syracuse University, Syracuse, New York, United States of America; 2 Department of Science Teaching, Syracuse University, Syracuse, New York, United States of America

## Abstract

Active learning methods have been shown to be superior to traditional lecture in terms of student achievement, and our findings on the use of Peer-Led Team Learning (PLTL) concur. Students in our introductory biology course performed significantly better if they engaged in PLTL. There was also a drastic reduction in the failure rate for underrepresented minority (URM) students with PLTL, which further resulted in closing the achievement gap between URM and non-URM students. With such compelling findings, we strongly encourage the adoption of Peer-Led Team Learning in undergraduate Science, Technology, Engineering, and Mathematics (STEM) courses.

## Abstract 2

This study shows that Peer-Led Team Learning drastically reduces the failure rate for underrepresented, minority undergraduate students in an introductory biology course.

Recent, extensive meta-analysis of over a decade of education research has revealed an overwhelming consensus that active learning methods are superior to traditional, passive lecture, in terms of student achievement in post-secondary Science, Technology, Engineering, and Mathematics (STEM) courses [1]. In light of such clear evidence that traditional lecture is among the least effective modes of instruction, many institutions have been abandoning lecture in favor of “flipped” classrooms and active learning strategies. Regrettably, however, STEM courses at most universities continue to feature traditional lecture as the primary mode of instruction.

Although next-generation active learning classrooms are becoming more common, large instructor-focused lecture halls with fixed seating are still the norm on most campuses—including ours, for the time being. While there are certainly ways to make learning more active in an amphitheater, peer-interactive instruction is limited in such settings. Of course, laboratories accompanying lectures often provide more active learning opportunities. But in the wake of commendable efforts to increase rigorous laboratory experiences at the sophomore and junior levels at Syracuse University, a difficult decision was made for the two-semester, mixed-majors introductory biology sequence: the lecture sections of the second semester course were decoupled from the laboratory component, which was made optional. There were good reasons for this change, from both departmental and institutional perspectives. However, although STEM students not enrolling in the lab course would arguably be exposed to techniques and develop foundational process skills in the new upper division labs, we were concerned about the implications for achievement among those students who would opt out of the introductory labs. Our concerns were apparently warranted, as students who did not take the optional lab course, regardless of prior achievement, earned scores averaging a letter grade lower than those students who enrolled in the lab. However, students who opted out of the lab but engaged in Peer-Led Team Learning (PLTL) performed at levels equivalent to students who also took the lab course [2].

Peer-Led Team Learning is a well-defined active learning model involving small group interactions between students, and it can be used along with or in place of the traditional lecture format that has become so deeply entrenched in university systems (Fig 1, adapted from [3]). PLTL was originally designed and implemented in undergraduate chemistry courses [4,5], and it has since been implemented in other undergraduate science courses, such as general biology and anatomy and physiology [6,7]. Studies on the efficacy of PLTL have shown improvements in students’ grade performance, attitudes, retention in the course [6–11], conceptual reasoning [12], and critical thinking [13], though findings related to the critical thinking benefits for peer leaders have not been consistent [14].

**Fig 1:**
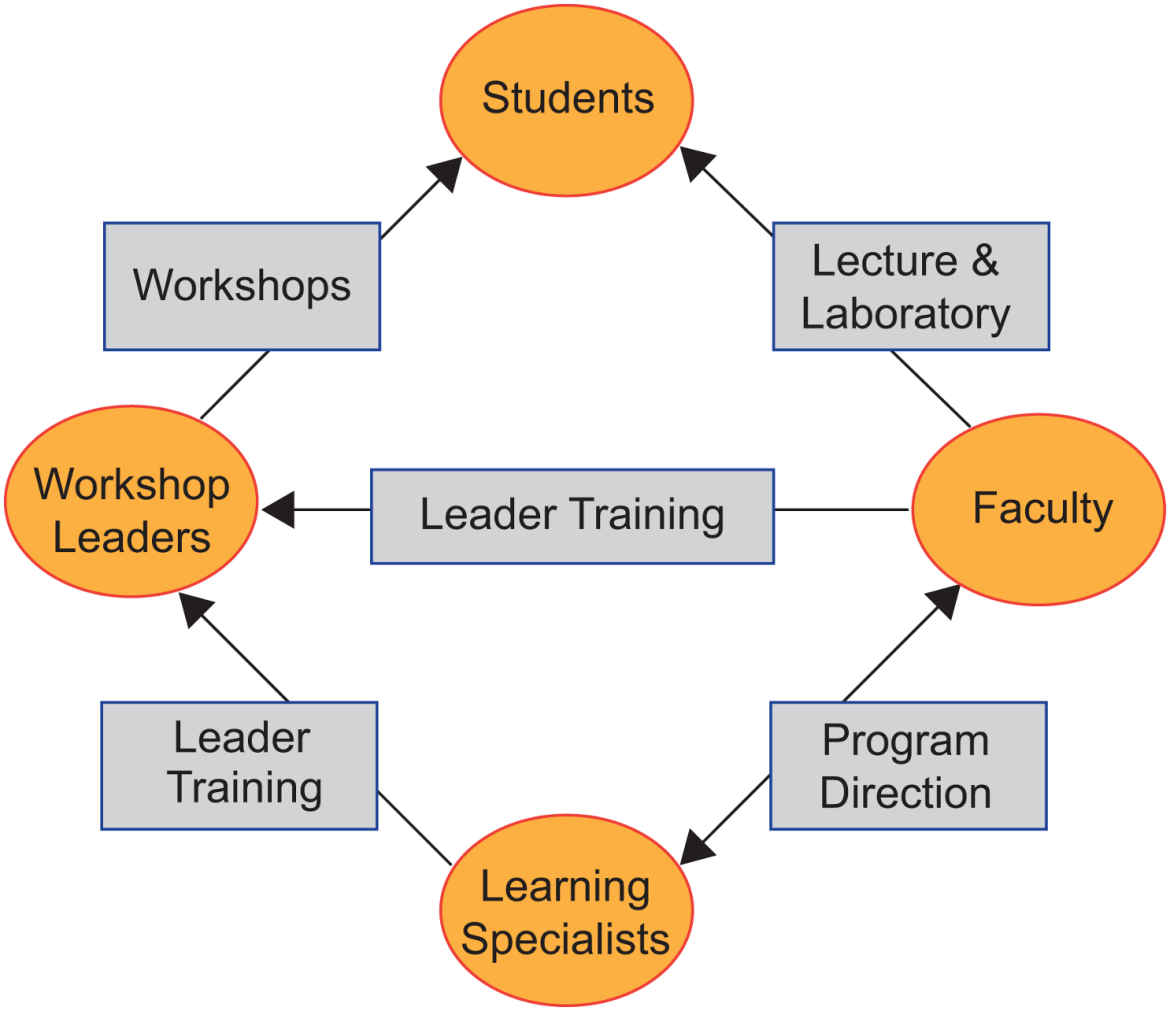
The PLTL model. In the PLTL workshop model, students work in small groups of six to eight students, led by an undergraduate peer leader who has successfully completed the same course in which their peer-team students are currently enrolled. After being trained in group leadership methods, relevant learning theory, and the conceptual content of the course, peer leaders (who serve as role models) work collaboratively with an education specialist and the course instructor to facilitate small group problem-solving. Leaders are not teachers. They are not tutors. They are not considered to be experts in the content, and they are not expected to provide answers to the students in the workshop groups. Rather, they help mentor students to actively construct their own understanding of concepts.

## PLTL and Underrepresented Minorities in STEM Fields

Along with our concern for student success in general, we have been especially focused on closing gaps for underserved groups within our student population. According to the National Academy of Sciences, efforts to increase the participation of underrepresented minorities (URMs) in STEM fields are essential to sustaining America’s research and innovation capacity [15]. Although members of minority groups have been earning an increasing number of post-secondary degrees since the 1990s, a substantially smaller proportion of minority students choose to pursue degrees in science and engineering than do students from groups that are traditionally well-represented in STEM [16]. Increasing recruitment of underrepresented minorities into STEM fields is a necessary effort, but retaining these students in STEM disciplines must also be a priority. Aside from the obvious social justice and equal access imperatives involved, the diversity of background and talent that students from underrepresented minority groups can bring to STEM fields is essential if we are to remain technologically innovative as global economic changes demand greater numbers of STEM professionals.

With high attrition rates of STEM majors in the United States, and even higher rates of underrepresented minorities leaving STEM disciplines at the undergraduate level, there has been a significant amount of research dedicated to interventions intended to increase the recruitment and retention of students in STEM disciplines. The literature reveals several factors that affect retention of underrepresented minorities in STEM, including mentoring [17], learning styles and strategies [17], earning a passing grade in gatekeeper courses [18], social networking [18], and reinforcing science identity [19].

Students who do not fare well in introductory STEM courses are far less likely to be recruited or retained in STEM majors, and when instruction involves only traditional lecture, there is a tendency for students to feel isolated and hopeless if they are not doing well [20]. The PLTL model incorporates a variety of learning styles and strategies, thus creating an environment conducive to social networking and reinforcement of science identity while developing students’ own understandings of scientific concepts in more accessible terms. We would therefore expect that URM students, in the context of such an environment, might achieve at higher levels than in traditional settings without PLTL. Indeed, Treisman [21] instituted a program based on small group interactions in the context of a large university mathematics course, with a goal of reducing academic isolation for underachieving students. Not only did this enhance learning and achievement, but it also reduced attrition. Among African American students in Treisman’s study, only 3% of the small group participants were unsuccessful in the course, compared to 40% of those who did not participate and 33% in the control group.

## Our Findings

Our experiences in using PLTL alongside the lecture hall approach in our introductory biology course have yielded exciting results. Among these are that retention in the course was higher for students who enrolled in PLTL, with those who did not attend PLTL sessions being significantly more likely to withdraw from the course (*x* ^2^ = 7.194, *n* = 479, df = 1, *p* = 0.007).

**Fig 2:**
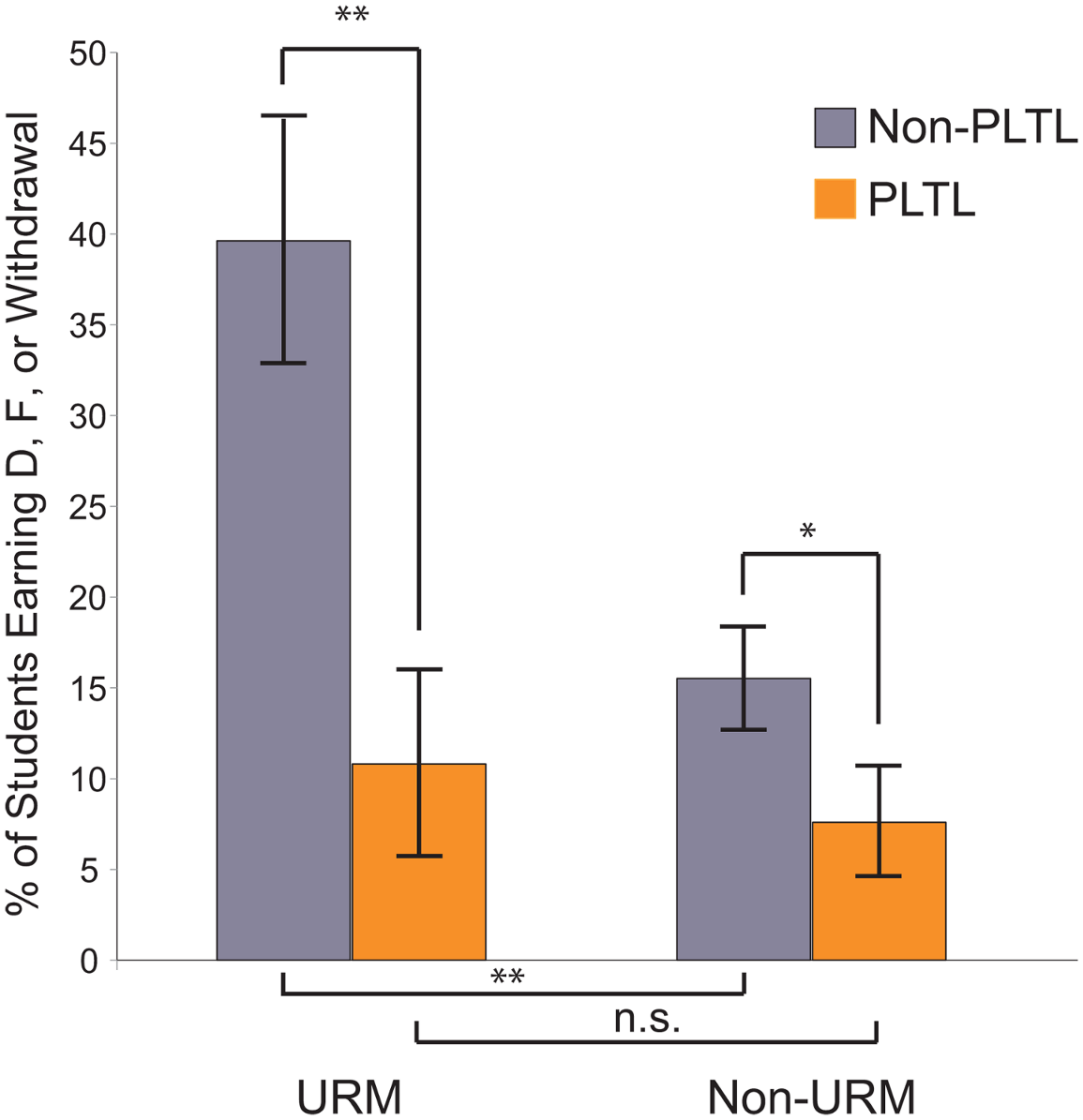
Achievement in introductory biology for URM and non-URM students with and without PLTL. Percent of students who earned a D, F, or withdrew (W) from the course. Values represent percent +/- standard error. Chi-square analyses reveal a significant gap between URM and non-URM students *(p* < 0.001) when these students do not participate in PLTL; this achievement gap is closed when students participate in PLTL *(p* = 0.272).

Perhaps even more encouraging is how PLTL appears to have influenced student achievement in the course, particularly for URMs (S1–S4 Tables). As shown in Fig 2, there was a dramatic and significant decrease—from nearly 40% down to about 15%—in the number of students earning Ds, Fs, or Withdrawing from the course (DFWs) among URMs who participated in PLTL (*x* ^2^ = 9.016, *n* = 90, df = 1, *p* = 0.003) and a smaller, but significant, decrease in DFWs for non-URMs as well (*x* ^2^ = 5.254, *n* = 251, df = 1, *p* = 0.022). The pronounced achievement gap between URMs and non-URMs was closed for URMs who sufficiently participated in PLTL. That is, the DFW rate was significantly higher for URMs than it was for non-URMs among those who did not engage in PLTL (*x* ^2^ = 14.157, *n* = 227, df = 1, *p* < 0.001), but not significantly different between URMs and non-URMs who did.

The results above are for all students whose URM or non-URM status could be determined *(n =* 479), regardless of concurrent enrollment in a lab course. There was no significant difference in prior achievement between students who opted out of PLTL or lab and those who engaged in these options (see S1 Text for additional methodological specifics). The laboratory component had been previously shown to be a factor in achievement [2]; however, we also found that DFW rates were lower among URMs who engaged in PLTL whether they were enrolled in the laboratory course (*x* ^2^ = 5.074, *n* = 69, df = 1, *p* = 0.024) or not (*x* ^2^ = 4.200, *n* = 21, df = 1, *p* = 0.040). Finally, we note that for URMs who did not participate in lab, half of those who did not engage in PLTL earned Ds, Fs, or withdrew from the course, while those who did engage in PLTL all completed the course and earned grades of C or higher.

## Conclusions, Recommendations, and Resources

Based on these data and on evidence from prior research, we are convinced that PLTL is effective in improving student achievement in introductory STEM courses, particularly for URM students. The drastic reduction in DFW rates among URM students and the closing of the achievement gap between URM and non-URM students are very compelling reasons to adopt the PLTL model, especially since significant gains were seen among non-URMs as well. The impact among students who are not concurrently enrolled in a lab course is a particularly important finding in the context of the biology program at our university, as several of the second-year courses in the biology major are not directly coupled with mandatory laboratory classes. What have we gained if we retain more diversity among life-science majors in their first year only to risk losing them as sophomores? It may be that a strong first year will help even the playing field when looking toward the second, so our future efforts will include tracking these students into upper-division courses as well as seeking to provide similar peer-interactive learning activities to students in all core courses in biology.

We also encourage other post-secondary educators to consider using PLTL, and many resources exist to help facilitate implementation in introductory biology and other STEM courses. Box 1 includes a number of helpful tools for beginning a PLTL program. We welcome inquiries regarding how we have undertaken these efforts as well as collaborations in research around this and other strategies in biology education.

**Box 1. Useful Resources**

**Books**

- Peer-Led Team Learning: A Guidebook. Gosser D, Cracolice M, Kampmeier J, Roth V, Strozak V, & Varma-Nelson P, eds. 2001. Upper Saddle River, NJ: Prentice Hall. ISBN-10: 0130288055
- Peer-led Team Learning: Origins, Research, and Practice. Gosser D. Ronkonkoma, NY: Linus Publications; 2015. ISBN-10: 1607975459
- Peer-Led Team Learning: A Handbook for Leaders. Roth V, Goldstein E, & Marcus G. Upper Saddle River, NJ: Prentice Hall; 2001. ISBN-10: 0131876058

**Free Online Resources**

- The Center for Peer-led Team Learning: https://sites.google.com/site/quickpltl
- Workshop Problem Sets: https://sites.google.com/site/quickpltl/workshop-materials
- Peer-Led Team Learning International Society: http://pltlis.org/

## Supporting Information

S1 Table Gender and ethnicity. (PDF)

S2 Table First generation students. (PDF)

S3 Table Prior final course grade performance. (PDF)

S4 Table SAT scores. (PDF)

S1 Text Implementation of PLTL. (PDF)

S1 Workshop Material PLTL first workshop session agenda. (PDF)

## Abbreviations

DFWs: Ds, Fs, or Withdrawing
PLTL: Peer-Led Team Learning
STEM: Science, Technology, Engineering, and Mathematics
URM: underrepresented minority
