# Supplementary material for: Peer-Led Team Learning Helps Minority Students Succeed
Source: PLoS Biol. 2016 Mar 9;14(3):e1002398. doi: 10.1371/journal.pbio.1002398 (PMC4784972; doi:10.1371/journal.pbio.1002398)
Supplement: S1 Table — (PDF) [file pbio.1002398.s001.pdf]

Table 1

Demographics for PLTL/Lab Groups (Gender and Ethnicity)

			Gender (%)		Ethnicity (%)				
Group		N	Male	Female	White	Black/African American	Hispanic/ Latino	Asian	Other
NonPLTL and NonLab	URM	15	1.5	2.9	0	1.8	0.6	0	2.1
	nonURM	47	5.6	8.2	11.4	0	0	2.3	0
PLTL Only	URM	6	0.6	1.2	0	0.9	0.3	0	0.6
	nonURM	10	1.2	1.8	2.3	0	0	0.6	0
Lab Only	URM	38	4.7	6.5	0	3.8	2.3	0	5.0
	nonURM	125	14.7	22.0	31.1	0	0	4.7	0.9
PLTL and Lab	URM	31	1.8	7.3	0	5	0.9	0	3.2
	nonURM	67	5.0	14.7	14.7	0	0	4.4	0.3
Total	URM	90	8.6	17.9	0	11.5	4.1	0	10.9
	nonURM	249	26.5	46.7	59.5	0	0	12.0	1.2
