# Supplementary material for: Peer-Led Team Learning Helps Minority Students Succeed
Source: PLoS Biol. 2016 Mar 9;14(3):e1002398. doi: 10.1371/journal.pbio.1002398 (PMC4784972; doi:10.1371/journal.pbio.1002398)
Supplement: S2 Table — (PDF) [file pbio.1002398.s002.pdf]

Table 2

Percent of First Generation Students in each PLTL/Lab Group

Group		N	First Generation (%)
Non PLTL and Non Lab	**URM**	13	0.3
	**nonURM**	38	0.9
PLTL only	**URM**	6	0.6
	**nonURM**	10	0.6
Lab Only	**URM**	38	3.8
	**nonURM**	125	7.9
PLTL and Lab	**URM**	31	4.1
	**nonURM**	67	5.0
Total	**URM**	88	8.8
	**nonURM**	240	14.4
