# Supplementary material for: Peer-Led Team Learning Helps Minority Students Succeed
Source: PLoS Biol. 2016 Mar 9;14(3):e1002398. doi: 10.1371/journal.pbio.1002398 (PMC4784972; doi:10.1371/journal.pbio.1002398)
Supplement: S3 Table — (PDF) [file pbio.1002398.s003.pdf]

Table 3

Final Grade Performance of each PLTL/Lab Group in First Semester Introductory Biology

Group		N	Prior Course Grade (%)
Non PLTL and Non Lab	**URM**	9	77.44 (6.41)
	**nonURM**	31	78.61 (10.09)
PLTL only	**URM**	3	82.09 (2.55)
	**nonURM**	8	84.97 (6.65)
Lab Only	**URM**	35	75.47 (8.50)
	**nonURM**	117	85.66 (7.55)
PLTL and Lab	**URM**	31	78.99 (7.10)
	**nonURM**	63	83.44 (7.99)
Total	**URM**	78	77.35 (7.72)
	**nonURM**	219	84.00 (8.35)

Note. Standard deviations in parentheses.
