# Supplementary material for: Peer-Led Team Learning Helps Minority Students Succeed
Source: PLoS Biol. 2016 Mar 9;14(3):e1002398. doi: 10.1371/journal.pbio.1002398 (PMC4784972; doi:10.1371/journal.pbio.1002398)
Supplement: S4 Table — (PDF) [file pbio.1002398.s004.pdf]

Table 4

Mean SAT scores for each PLTL/Lab Group

Group		N	SATV	SATM	SAT Total
Non PLTL and Non Lab	**URM**	14	528.57 (74.20)	527.14 (88.36)	1055.71 (149.09)
	**nonURM**	42	597.62 (75.09)	571.19 (57.81)	1168.81 (116.60)
PLTL only	**URM**	5	534.00 (65.04)	502.00 (77.59)	1036.00 (97.88)
	**nonURM**	6	585.00 (21.68)	611.67 (75.48)	1196.67 (91.36)
Lab Only	**URM**	37	535.68 (74.59)	526.49 (58.03)	1062.16 (118.21)
	**nonURM**	113	587.43 (72.70)	609.47 (70.00)	1196.90 (126.65)
PLTL and Lab	**URM**	26	505.38 (59.68)	506.15 (67.89)	1011.54 (98.86)
	**nonURM**	54	557.96 (59.19)	595.74 (61.48)	1153.70 (98.75)
Total	**URM**	82	524.76 (69.62)	518.66 (67.66)	1043.41 (117.40)
	**nonURM**	215	581.95 (70.25)	598.60 (67.01)	1180.56 (118.18)

Note. Standard deviations in parentheses.
