# Supplementary material for: Peer-Led Team Learning Helps Minority Students Succeed
Source: PLoS Biol. 2016 Mar 9;14(3):e1002398. doi: 10.1371/journal.pbio.1002398 (PMC4784972; doi:10.1371/journal.pbio.1002398)
Supplement: S1 Text — (PDF) [file pbio.1002398.s005.pdf]

Supporting Information

Implementation of PLTL

In implementing PLTL to the introductory biology course we adhered to the workshop model as described by Gosser [1]. A learning specialist (in our case, a Postdoctoral scholar with training in science education) recruited students who had been previously successful in the course to be peer mentors (success is generally defined as having earned an “A” or “B”). Students who were interested and available to serve as peer mentors were awarded academic credit in lieu of monetary payment and were thus enrolled in a leader training course that met once a week for training in learning theory, group leadership methods, and conceptual content. In addition to attending the training class once a week, peer mentors led a one-hour workshop session with eight or fewer students taking introductory biology.

Because this was the first time offering PLTL Workshop sessions in introductory biology at our university, students were offered a minimal amount of extra credit to attend the sessions in addition to lecture. This extra credit was disregarded in our analyses of student achievement. During an introductory biology lecture at the beginning of the semester, students were told about the opportunity to participate in small group problem-solving sessions each week with a peer leader who was already successful in the course. An email with sign-up instructions and the same information about PLTL presented in lecture was also sent out to the students. Although students voluntarily opted to participate in the peer-led workshop sessions, enrollment based on prior student-student friendship or student-leader friendship was minimized, as well as discrimination, by posting the available workshop times without the peer leaders’ names or the names of other enrolled students. It is important to note that students who opted to engage in PLTL did not differ statistically from those who did not participate in PLTL in terms of prior achievement in the previous semester.

During the first training session, peer mentors were provided with a description of the PLTL model, a first workshop agenda (S1 Workshop Material), and a peer leader handbook [2]. Successive training sessions

included activities related to the weekly reading from the handbook, debriefing on the previous week's workshop session, and collaboration of the peer mentors on weekly problem sets that coincided with the lecture content for the week.

During the workshop sessions, peer mentors guided their students through problem sets that were created through the collaboration of the learning specialist and course instructor (Workshop materials can be found here: https://sites.google.com/site/quickpltl/workshop-materials or via the link in Box 1 of the main article.). Weekly problem sets included challenging, conceptually based problems and corresponded to common topics for introductory biology and included but were not limited to such topics as photosynthesis, cellular respiration, plant reproduction and development, animal structure, and animal nutrition.

To obtain feedback on the effectiveness of the sessions, peer mentors completed weekly journals in which they reflected on the session. These reflections included feedback on group dynamics, understanding of material by the students, difficulties encountered, methods/strategies used, and types of problems that were beneficial or not-so-beneficial to the understanding of specific concepts.

The statistical test for course retention was performed using Pearson chi-squared analyses including all students whose URM/Non-URM status was known based on institutional data collected during the admissions process (N=479). In subsequent analyses, to look at the effect on PLTL between URM and non URM students, we defined adequate PLTL participation as previously determined [3] and used Pearson chi-squared tests.

Thorough descriptions of our implementation of PLTL can be found in [3] and [4].

Ethics Statement

Data reported in this manuscript were collected according to protocols approved by the Syracuse University Institutional Review Board. Prior to collecting the data, participants were provided with an informed consent form via email. Participants were able to have their data excluded from the research

dataset at any time, without penalty, and without the knowledge of the researchers by contacting a non-instructor/non-researcher third party charged with managing the data. For privacy protection, each voluntary participant was assigned a unique identification number by the third party administrator which could be matched across data collection instruments.

References

1. Gosser D. The peer led team learning workshop model. In: Peer-Led Team Learning: A Guidebook, ed. D Gosser, M Cracolice, J Kampmeier, V Roth, V Strozak, P Varma-Nelson, Upper Saddle River, NJ: Prentice Hall; 2001. pp. 1-12.
2. Roth V, Goldstein E, Marcus G. Peer-Led Team Learning: A Handbook for Leaders, Upper Saddle River, NJ: Prentice Hall; 2001.
3. Snyder JJ, Carter BE, Wiles JR. Implementation of the peer led team learning instructional model as a stopgap measure improves student achievement for students opting out of laboratory. CBE Life Sci Edu. 2015;14: 1-6.
4. Snyder JJ, Wiles JR (2015) Peer led team learning in introductory biology: Effects on peer leader critical thinking skills. PLoS One. 2015;10(1): e0115084. doi:10.1371/journal.pone.0115084
