# Supplementary material for: Peer-Led Team Learning Helps Minority Students Succeed
Source: PLoS Biol. 2016 Mar 9;14(3):e1002398. doi: 10.1371/journal.pbio.1002398 (PMC4784972; doi:10.1371/journal.pbio.1002398)
Supplement: S1 Workshop Material — (PDF) [file pbio.1002398.s006.pdf]

The First Peer Led Team Learning Workshop Session Agenda

1. INTRODUCTION OF YOURSELF: Take a few moments to introduce yourselves to your students. Tell them what your major is, when you took BIO 123, what your future plans are such as pre-med, etc., and any other information that you may like to share with them related to academics.
2. STUDENT INTRODUCTIONS: Have your students introduce themselves to you and to each other. You can do this in any way that you would like. You may want to have them pair up and introduce themselves to each other, and then have them introduce their partner to the rest of the students. Or, you may have other icebreaker activities that you have done or would like to do with your students. Whatever you choose to do is fine, as long as you try to get the group to interact with each other a bit!
3. DESCRIPTION OF PLTL SESSIONS: Following introductions, you will want to describe how the Workshop sessions will run and what your role as a leader will be. Explain to the students that they will be working on problem sets as a group and that you are there to guide and help them through the material. Emphasize that you are not there to just give the answers, rather you will help them find good problem solving approaches to determine answers to the problems. Do this in a way that you are comfortable with!
4. EXPECTATIONS: You will need to be direct in explaining the expectations for the sessions. Tell the students that they are expected to be on time and actively participating in the problem sets to obtain the extra credit points. Also, emphasize that they are expected to be there for the full hour to receive credit. Students are also expected to print and bring the problem sets not completed with them to class. They will access the problem sets via blackboard.
5. PROBLEM SET: Have students begin to work on the problems together. As discussed in class, you can do the problems in whatever order you (and your students) decide, and you can break students up according to what will work best for the number of students you have.

Keep in mind that this is the first week of your session and that many students may not attend this week. They will likely be in the process of signing up for a time and so on. If no one has shown up for your session, please wait at least 15 minutes to see if any stragglers end up coming. If not, you are free to go. Have a great first week!
